# Evaluation of Serum Interleukin 6, Tumor Necrosis Factor-Alpha, and Interferon-Gamma Levels in Relation to Body Mass Index and Blood Pressure in HIV Seropositive Pregnant Women Coinfected with Malaria

**DOI:** 10.1155/2020/2424802

**Published:** 2020-10-28

**Authors:** Ikechukwu Uzoma Chukwuagwu, Nkiruka Rose Ukibe, Innocent Ikechi Ogbu, Charles German Ikimi, Victoria Ogechi Agu, Ofia Anya Kalu, Solomon Nwabueze Ukibe, Joseph Chimezie Awalu

**Affiliations:** ^1^Department of Medical Laboratory, Faculty of Health Sciences and Technology, Nnamdi Azikiwe University, Nnewi Campus, PMB 5025, Nnewi, Anambra State, Nigeria; ^2^Department of Biochemistry, Faculty of Science, Federal University of Otuoke, Yenagoa, Beyalsa State, Nigeria; ^3^Department of Medicine, Faculty of Medicine, Nnamdi Azikiwe University, Nnewi Campus, PMB 5025, Nnewi, Anambra State, Nigeria; ^4^Department of Medical Microbiology, Faculty of Medicine, Nnamdi Azikiwe University, Nnewi Campus, PMB 5025, Nnewi, Anambra State, Nigeria

## Abstract

Malaria and HIV are leading causes of morbidity and mortality, particularly in sub-Saharan Africa. Both diseases are highly endemic and have a wide geographic overlap with severe impact on pregnancy. This was a case-control study designed to evaluate the levels of interleukin -6 (IL-6), tumor necrosis factor-alpha (TNF-*α*), and interferon-gamma (IFN-*γ*) and their relationship with some anthropometric indices such as body mass index (BMI) and blood pressure in HIV-malaria coinfected women attending antenatal clinic at Nnamdi Azikiwe University Teaching Hospital (NAUTH), Nnewi, Nigeria. 122 pregnant women and 30 nonpregnant women (control) aged between 18 and 42 years were recruited for the study. Screening of HIV antibodies was done using a national algorithm. Peripheral malaria was determined using rapid detection and the Giemsa stain technique. Cytokines were assayed using the enzyme-linked immunosorbent assay technique. HIV-malaria coinfected pregnant women showed significantly higher levels of IL-6, IFN-*γ*, TNF-*α*, and blood pressure with reduced BMI value compared with HIV seronegative pregnant and nonpregnant control participants (*p* ≤ 0.001, respectively). The findings indicated significant cytokine imbalance which suggests an active inflammatory process and reduced cellular immunity. The increased BMI and blood pressure level observed indicate overweight and possible hypertension which could subsequently lead to preeclampsia and other adverse pregnancy outcomes.

## 1. Introduction

Human immunodeficiency virus (HIV) and *Plasmodium falciparum* malaria are two of the major lethal infectious diseases in sub-Saharan Africa which can infect pregnant women with detrimental effects to the mother as well as the fetus [1, 2]. Little is known about the immunological implication of their coinfection [3], especially with regard to the various challenges encountered in both investigation and treatment of patients.

HIV-malaria coinfection has been characterized by the activation of several inflammatory pathways with the release of cytokines, some of which can be detrimental to the host cell and contribute to high morbidity and mortality especially during pregnancy [4]. This can easily occur due to reduced immunity [5] which also enhances coinfections such as malaria especially in places of high endemic transmission [6]. Reports have shown evidence of increased risk of placental malaria in HIV-infected pregnant women [7], which can grossly reduce their immunity to malaria infection leading to modulation of circulating cytokines [8] with synergistic exposure to adverse pregnancy outcomes [9].

Chronic nonresolving inflammation will further contribute to immunodeficiency through mechanisms such as immune exhaustion [10]. Certain factors like body weights and changes in blood pressure play important roles in this interplay of biochemical reactions. Pregnancy is a particular period during which the body undergoes physiological adjustments, one of them being the modulation of adaptive, proinflammatory immune responses to ensure fetal survival [11]. Cytokines are identified to be of particular importance in mediating communications between the conceptus and maternal cells, particularly the uterine epithelium and infiltrating leukocytes, both prior to implantation and as the placenta develops [12]. However, abnormal secretion of these cytokines has been implicated in placental malaria [13, 14] especially in HIV seropositive pregnant women coinfected with malaria parasitemia. The present study, therefore, seeks to examine the association between cytokines IL- 6, TNF-*α*, IFN-*γ*, and some anthropometric indices such as body mass index and blood pressure among pregnant women with HIV-malaria coinfection at NAUTH, Nnewi, Nigeria.

## 2. Materials and Methods

### 2.1. Study Design

This is a case-control study designed to assess the levels of maternal inflammatory markers (IL-6, IFN-*ɣ*, and TNF-*α*) and possible association with body mass index and blood pressure in naïve (newly diagnosed) HIV seropositive pregnant women with malaria coinfection attending the antenatal clinic at Nnamdi Azikiwe University Teaching Hospital, in Nnewi (NAUTH), Nigeria. A total of 152 female participants including 122 pregnant women were recruited for this study. The study comprised randomly selected HIV seropositive pregnant women with malaria parasitemia (32), HIV seropositive pregnant women without malaria parasitemia (30), malaria-infected pregnant women without HIV infection (30), pregnant women without HIV and malaria parasitemia (30) as control, and nonpregnant women without HIV and malaria infection (30) as another control. All HIV seropositive pregnant women were yet to commence antiretroviral therapy (naïve). All subjects were screened for HIV seropositivity and malaria parasitemia. Screening for HIV antibodies was by the DETERMINE and STAT-PAK and confirmed with UNIGOLD. Pregnancy testing was done by human chorionic gonadotropin (HCG) one step pregnancy test strip, and their gestational period was between 11 and 28 weeks. Peripheral malaria was determined from maternal venous blood by Rapid Detection Technique (RDT) (2SD). Giemsa stains of thin and thick blood smears were assayed by microscopy to confirm malaria results.

A well-structured questionnaire was administered to each participant to obtain the history of their pregnancy and other biodata. Medical history such as TB and hepatitis status was obtained from their medical records. The participants were aged between 18 and 42 years. Levels of cytokines were assayed using enzyme-linked immunosorbent assay (ELISA) technique while blood pressure and body mass index were determined using standard measurements and calculations.

### 2.2. Study Site

The study was conducted at Nnamdi Azikiwe University Teaching Hospital (NAUTH) in Nnewi, Anambra State, Nigeria. Laboratory analysis of cytokines was carried out at the Chemical Pathology Laboratory of Nnamdi Azikiwe University Teaching Hospital, Nnewi.

### 2.3. Subject Recruitment

A purposive sampling technique was employed. The participants were pregnant women visiting the PMCT clinic at NAUTH between December 2017, and April 2018, who voluntarily agreed to participate and were subsequently enrolled in the study.

### 2.4. Ethical Considerations

In line with the Helsinki Declaration, approval for this study was obtained from the Human Research Ethics Committee of the Nnamdi Azikiwe University Teaching Hospital, Nnewi, Anambra State (NAUTH/CS/66/VOL.10/194/2017/104). The procedures were explained to the subjects and written informed consent was obtained from each subject before enrolling in the study. They were assured of the confidentiality of the information obtained from them during and after the study.

### 2.5. Inclusion and Exclusion Criteria

Pregnant women (11–28 weeks) aged between 18 and 42 years were included in the study. HIV-infected pregnant participants with/without malaria infection and HIV seronegative nonpregnant participants with/without malaria infection were also included. However, pregnant women less than 18 and above 42 years were excluded from the study. Participants who had tuberculosis and hepatitis were excluded from the study. Participants who are active smokers, alcoholics, and diabetic patients were excluded.

### 2.6. Sample Collection

Five milliliters of venous blood was collected from each of the subjects and dispensed into a well-labeled plain container and allowed to clot. The sample was centrifuged at 252 ×g for 10 min. The serum was separated and dispensed into plain containers and stored at −80°C in the specialized laboratory of NAUTH, Nnewi, until assayed for IL-6, TNF-*α*, and IFN-*ɣ* in the Chemical Pathology Laboratory Department, Nnamdi Azikiwe University Teaching Hospital (NAUTH) Nnewi, Anambra State, Nigeria.

### 2.7. Anthropometric Measurements

The physical measurements (body weight and height) were taken using a standard beam balance scale and a stadiometer, respectively; participants were advised to wear light clothing with no shoes for an accurate measurement. Values obtained were used to calculate BMI (kg/m^2^) [weight (kg)/(height)^2^ (m^2^)]. Blood pressure (systolic and diastolic) was also measured using a standard clinical mercury sphygmomanometer.

### 2.8. Methods

HIV-1/2 assays were done according to the national algorithm using HIV-1/2 assay [15], Uni-gold HIV test [16], and Stat-pak HIV test [17].

Malaria parasite screening was done using a rapid detection test for *Plasmodium falciparum* malaria antigen as described by Murray and Gresser [18] and Giemsa stained thick and thin blood film for microscopic detection of *P. falciparum* parasites as described by WHO [19].

Human TNF-*α* immunoassay was determined using an enzyme-linked immunosorbent assay (ELISA) method according to Megnekov et al. [20] while interleukin 6 (IL-6) and IFN-*γ* were done as described by Suguitan et al. [21].

### 2.9. Statistical Analysis

Statistical package for social sciences (SPSS) version 22 was used for the statistical analysis. The data generated was analyzed using the analysis of variance (ANOVA) to compare more than two groups per variable and Student's *t*-test for two independent variables. The Pearson correlation was used to correlate different parameters. GraphPad Prism version 7.04 was used for grouped data comparison. Values were considered statistically significant if *p* value ≤ 0.05.

## 3. Results

### 3.1. Mean Values of Some Anthropometric Variables in HIV Seropositive Pregnant Women and Control Participants with/without Malaria Infection

The mean BMI value was compared between the test groups and the control group. BMI was significantly lower in HIV seropositive pregnant women with/without malaria parasitemia (26.18 ± 2.59, 26.85 ± 3.10) compared with HIV seronegative pregnant women with malaria coinfection (28.59 ± 3.70) but higher than their nonpregnant counterpart (24.66 ± 1.38) (*p* ≤ 0.001, respectively). The between-group comparison showed that BMI was significantly lower in HIV seropositive pregnant women with malaria parasitemia (26.18 ± 2.59) compared with HIV seronegative pregnant women with malaria coinfection (28.59 ± 3.70) (*p*=0.020). Furthermore, the mean BMI value was significantly higher in HIV seronegative pregnant women with/without malaria (28.59 ± 3.70, 27.21 ± 3.41) compared with their control counterpart (24.66 ± 1.38) (*p* ≤ 0.001).

The mean DBP was compared between the test groups and the control group. DBP was significantly higher in HIV seropositive pregnant women with malaria coinfection (85.53 ± 9.90), HIV seronegative pregnant women with malaria parasitemia (82.96 ± 9.86) compared with their corresponding groups without malaria parasitemia (79.83 ± 9.30, 77.60 ± 5.75), and control subjects (73.83 ± 3.55) (*p* ≤ 0.001). The between-group comparison showed that the mean DBP value was significantly higher in HIV seropositive pregnant women without malaria parasitemia (79.83 ± 9.30) and HIV seronegative pregnant women with malaria parasitemia (82.96 ± 9.86) compared with control (73.83 ± 3.55) (*p*=0.005,  *p* ≤ 0.001, respectively). The mean DBP value was significantly higher in HIV seronegative pregnant women with malaria parasitemia (82.96 ± 9.86) compared with their counterparts without malaria parasitemia (77.60 ± 5.75) (*p*=0.012).

**Figure 1:**
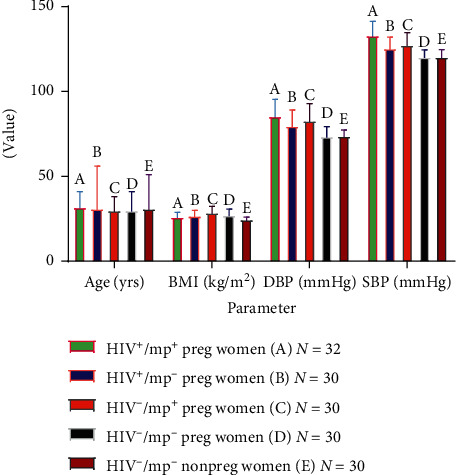
Comparison of the mean (±SD), age, body mass index, and blood pressure levels in the test group and control group. Key: BMI in A and B vs C and D (*p* ≤ 0.001 respectively). BMI in A vs C (*p*=0.020). BMI in A and B vs E (*p* ≤ 0.001). DBP in A and C vs B, D, and E (*p* ≤ 0.001 respectively). B and C vs E (*p*=0.005,  *p* ≤ 0.001 respectively). C vs D (*p*=0.012). SBP in A, B, C, and D vs E (*p* ≤ 0.001). A vs B (*p* ≤ 0.001).C vs D (*p* ≤ 0.001).

The mean SBP value was compared between the test groups and the control group. The mean SBP value was significantly higher in HIV seropositive pregnant women with/without malaria parasitemia (133.18 ± 8.18, 125.4 ± 6.71), HIV seronegative pregnant women with/without malaria parasitemia (127.56 ± 7.14, 120.43 ± 4.21) compared with control participants (120.43 ± 4.24) (*p* ≤ 0.001). Similarly, SBP was significantly higher in HIV seropositive pregnant women with malaria parasitemia (133.18 ± 8.18) compared with the corresponding group without malaria (125.4 ± 6.71) (*p* ≤ 0.001). SBP was significantly higher in HIV seronegative pregnant women with malaria (127.56 ± 7.14) than in HIV seronegative pregnant women without malaria parasitemia (120.43 ± 4.21) (*p* ≤ 0.001) (Figure 1).

### 3.2. Levels of Serum IL-6, TNF-*α*, and IFN-*ɣ* in HIV Seropositive Pregnant Participants with Malaria Coinfection and Control Participants

The mean levels of IL-6 were compared between the test groups and the control group. The mean IL-6 level was significantly lower in HIV seropositive pregnant women without malaria parasitemia (1.83 ± 0.55), HIV seronegative pregnant women with malaria parasitemia (1.80 ± 0.57) compared with HIV seropositive pregnant women with malaria (2.45 ± 1.05), HIV seronegative pregnant women without malaria parasitemia (2.34 ± 0.90), and nonpregnant HIV seronegative women without malaria (2.17 ± 1.04) (*p*=0.008). The between-group comparison showed that IL-6 level was significantly higher in HIV seropositive pregnant women with malaria parasitemia (2.45 ± 1.05) when compared with HIV seropositive pregnant women without malaria (1.83 ± 0.55) and HIV seronegative pregnant women with malaria (1.80 ± 0.57) (p=0.005, 0.003, respectively). On the other hand, the IL-6 level was significantly lower in HIV seropositive pregnant women without (1.83 ± 0.55) and HIV seronegative pregnant women with malaria (1.80 ± 0.57) compared with HIV seronegative pregnant women without malaria parasitemia (2.34 ± 0.90) (*p*=0.023, 0.016, respectively).

The mean level of IFN-*ɣ* was compared between the test groups and the control group. The mean IFN-*ɣ* level was significantly higher in HIV seropositive pregnant women with malaria coinfection (103.97 ± 16.67) compared with HIV seropositive pregnant women without malaria parasitemia (75.82 ± 9.50), HIV seronegative pregnant women with malaria parasitemia (92.28 ± 11.59), HIV seronegative pregnant women without malaria parasitemia (83.28 ± 10.40), and control participants (58.71 ± 6.57) (*p* ≤ 0.001).

The mean level of IFN-*ɣ* was significantly lower in HIV seropositive pregnant women without malaria parasitemia (75.82 ± 9.50) compared with HIV seronegative pregnant women with/without malaria parasitemia (92.28 ± 11.59, 83.28 ± 10.40) (*p*=0.014, respectively). IFN-*ɣ* level was significantly higher in HIV seronegative pregnant women with malaria parasitemia (92.28 ± 11.59) compared with their corresponding group without malaria parasitemia (83.28 ± 10.40) and control group (58.71 ± 6.57) (*p*=0.003, ≤0.001 respectively). A similar observation was made between HIV seronegative pregnant women without malaria parasitemia (83.28 ± 10.40) and the control group (58.71 ± 6.57) (*p* ≤ 0.001).

**Figure 2:**
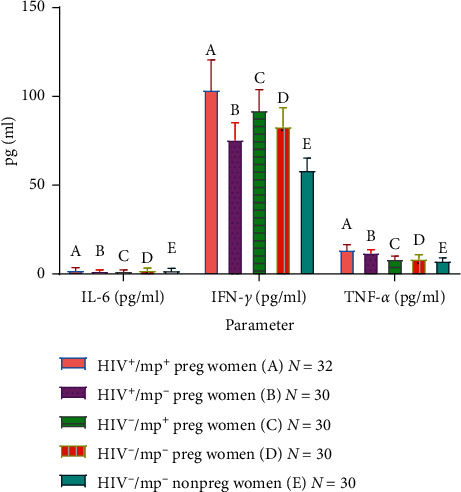
Comparison of mean (±SD) plasma levels of IL-6, IFN-*γ*, and TFN-*α* in test group and control group. Key: IL-6 in B and C vs A, D, and E (*p*=0.008). IL-6 in A vs B and C (p=0.005,  *p* ≤ 0.003, respectively). IFN-*γ* in A vs B, C, D, and E (*p* ≤ 0.001). IFN-*γ* vs CD (*p*=0.014). IFN-in C vs D and E (*p*=0.003,  *p* ≤ 0.001. D vs E (*p* ≤ 0.001). TNF-*α* in A and B vs E (*p* ≤ 0.001).

The mean level of TNF-*α* was significantly higher in all the test groups (13.79 ± 2.66, 12.12 ± 1.64, 8.69 ± 1.42, 8.77 + 2.1) compared with the control group (7.53 ± 1.42) (*p* ≤ 0.001) except between HIV seronegative pregnant women with malaria parasitemia (8.69 ± 1.42) and their counterpart without malaria parasitemia (8.77 + 2.1) (*p*=0.877) (Figure 2).

### 3.3. Correlation of Inflammatory Markers with Some Anthropometric Parameters in HIV Seropositive Pregnant Participants with/without Malaria and Control Participants

The Pearson correlation was applied to the parameters under study. There was a positive correlation between IL-6 and IFN-*ɣ* in HIV seropositive pregnant women with malaria coinfection (*r* = 0.479, *p*=0.006). Similarly, there was a positive correlation between IFN-*ɣ* and BMI in nonpregnant HIV seronegative women without malaria parasitemia (*r* = 0.433, *p*=0.017). However, a negative correlation existed between TNF-*α* and SPB in HIV seropositive pregnant women without malaria parasitemia (*r* = −0.36, *p*=0.048) (Table 1).

## 4. Discussion

HIV and malaria coinfection is associated with the activation of several inflammatory pathways, resulting in systemic and local inflammatory responses that involve the production of a wide range of cytokines. The present study showed that serum levels of IL- 6, TNF-*α*, and IFN-*γ* were significantly elevated in HIV seropositive pregnant participants with malaria coinfection. The elevation was more marked in pregnant participants with malaria parasitemia in comparison with HIV seropositive pregnant participants. Fievet et al. [12] and Onifade et al. [22] reported similar findings. The overtly amplified condition suggests a subtle interplay between the already compromised placental and peripheral tissue integrity due to the combined effects of malaria parasitemia and viral load associated with HIV infection. The increased production of proinflammatory cytokine in HIV and malaria coinfected pregnant women was also attributed to a compromised immune response against free radical induced stress [23], thereby predisposing them to internal tissue/cell damage.

The significant increases in IL-6 and TNF-*α* level observed in the HIV seropositive pregnant participants with malaria coinfection could be indicative of viral replication. The exaggeration may be in response to the immunological regulation in pregnancy [24]. A positive correlation between IL- 6 and TNF-*α* was also observed in HIV seropositive pregnant women with malaria coinfection. Further studies have indicated that acute malaria can also increase cytokine production which could subsequently lead to viral replication and immunological interactions [9, 25]. In HIV infection, molecular redox balance is disturbed in CD4^+^ T cells which are accompanied by severe immune dysfunction thus, potentiating HIV replication. Malaria infections seem to accelerate the degree of reduction of antioxidants in HIV-infected participants. Various studies in Nigeria have documented a high prevalence of malaria among HIV-infected individuals [26, 27], showing the impact of the combined effect of malaria and HIV in areas with generalized HIV epidemic and malaria-endemic transmissions. However, routine administration of improved antioxidant supplemented antimalaria drugs would inhibit the in vitro and in vivo growth and development of the malaria parasite [28].

This study showed an increased level of IFN-*ɣ* in HIV seropositive pregnant women with malaria coinfection or as single infection with malaria as compared with HIV seropositive women without malaria. Djontu and colleagues documented a similar report [29]. The assertion is that the increased level of systemic IFN-*ɣ* is in response to increased parasitemia. The increased level has been associated with the predominance of CD8+ thereby, inhibiting CD4+ production as seen in HIV-infected pregnant women with malaria coinfection compared to HIV seropositive pregnant participants without malaria. This action is in response to an attempt to ensure parasite clearance, thereby, showcasing a level of protective immunity in pregnant women [30]. The elevated level of IFN-*ɣ* among pregnant women with malaria parasitemia suggests that IFN-*ɣ* is a major mediator in the host responses to systemic *Plasmodium falciparum* malaria in our locality.

The loss of body mass was very significant among HIV seropositive pregnant women with or without malaria coinfection. The observation is in contrast with some previous studies [31, 32]. Consequently, among HIV-infected persons, secondary infections or coinfection were also signiﬁcant predictors of low gestational weight gain [33], possibly through secretion of proinﬂammatory cytokines. Loss of fat mass due to infection during pregnancy might represent a decrease in the available substrate for fetal growth. In addition, weight loss could be a risk factor for chorioamnionitis through impairment in speciﬁc immune responses [34], which increases the risk of stillbirth and preterm delivery. In contrast, a previous study has shown that the BMI of people living with HIV/AIDS does not differ from that of the general population [35]. The differences observed in this study might be that some HIV seropositive pregnant women might be under stress compounded by their poor nutritional and socioeconomic status. The body mass index (BMI) is an anthropometric measurement for defining body composition and nutritional status. Initially, BMI was used as a measure of obesity in developed countries, but it is now applied to define underweight and overweight adults in countries throughout the world [36]. In developed countries, the poor nutritional status of women as defined by a low BMI has a negative effect on pregnancy outcomes, particularly low infant birth weight and preterm delivery [37].

Similarly, it was observed that the blood pressure of HIV seropositive pregnant women was significantly higher than that of control pregnant women. The observation suggests that HIV-seropositive pregnant women might have tendency to develop hypertension and possibly preeclampsia. Studies have reported a higher prevalence of hypertension in the HIV-infected population than in the HIV-uninfected population [38, 39]. Increasing blood pressure could be related to HIV-specific factors such as lipodystrophy, atherogenesis, and cytokines activity [40]. Infection with the human immunodeficiency virus type 1 in pregnant women represents an independent risk factor for maternal mortality, stillbirth, and intrauterine growth restriction (IUGR) [41]. Immune hyperactivity to paternal antigens has been hypothesized to play a role in the development of hypertension and the immunosuppression caused by HIV could temper the immune response at the placental site and reduce placental vasoconstriction [42]. This potential protection may be a function of the intensity of immunosuppression and may depend on the severity of HIV disease and the use of antiretroviral therapy. Little information is available regarding hypertension during pregnancy, its prevalence, risk factors, and the effects of blood pressures on pregnancy outcomes among HIV-infected women in sub-Saharan Africa.

Notably, just as with the case of HIV seropositive pregnant women without malaria parasitemia in this study, there is a significant increase in the diastolic and systolic blood pressure in HIV seronegative pregnant women with malaria parasitemia when compared with controls. Ndao and others reported similar findings [43]. In areas of stable endemic malarial transmission such as in the study area, *P.falciparum* infection during pregnancy is usually asymptomatic [43]. It is characterized by the sequestration of parasites in the placenta. Massive sequestration of parasites in the placenta leads to placental ischemia, increases the production of proinﬂammatory cytokines, and increases endothelial dysfunction [44]. In a normal pregnancy, the earliest stages of development take place in a low oxygen environment-tissue hypoxia [45]. Tissue hypoxia is known to promote the release of ROS that are potentially damaging to the cardiovascular system [46]. Pathological stress is mainly due to disease conditions including malaria, helminthiasis, HIV, diabetes, and hypertension [47]. The effects of malaria in pregnancy have been well described. It has been documented that malarial infection during pregnancy is a major cause of low birth weight babies and maternal anemia [48, 49]. Additionally, in sub-Saharan Africa, the rates of both preeclampsia and malaria increase during the rainy season. Some studies have observed a higher incidence of preeclampsia during the peak of malarial transmission during the rainy season [50]. In an earlier study, a strong association was found between active malarial infection (identiﬁed by peripheral parasitemia) and bilateral notching of the uterine artery Doppler waveforms in late pregnancy (32–35 weeks' gestation) as observed in women with preeclampsia [51].

As has been described earlier and as a consequence of coinfection, HIV seropositive pregnant women have a higher risk of developing severe malaria infection particularly in malaria-endemic areas with attendant immune dysregulation [38, 52]. Proinflammatory cytokines such as TNF-*α* released by cells of the immune system can activate the endothelium, increasing the expression of surface adhesion molecules that contribute to the sequestering of infected erythrocytes, adhesion of platelets, and mononuclear cells [53]. This mechanism of inflammation and sequestration gives rise to tissue stress and a significant rise in diastolic and systolic blood pressure as confirmed in this study.

## 5. Conclusion

HIV and malaria coinfection impacts serious significant changes in the levels of maternal inflammatory markers such as IL-6, TNF-*α*, and IFN-*ṿ* in pregnant women. HIV and malaria coinfection is associated with strong upregulation of proinflammatory cytokines which may be a result of strong CD4+ cell activation which then confers a bidirectional burden of both diseases in pregnancy. There is a strong relationship between the anthropometric indices (SBP and BMI) and cytokines (TNF-*α* and IFN-*ṿ*) in HIV seropositive pregnant women with malaria coinfection. The cytokine imbalance suggests an active inflammatory process and reduced cellular immunity. The increased BMI and blood pressure level observed indicates possible hypertension which could subsequently lead to preeclampsia and other adverse pregnancy outcomes. Frequent evaluation of these cytokines could serve as a diagnostic adjunct marker in HIV seropositive pregnant women with malaria coinfection especially in areas of high endemic transmission.

## Figures and Tables

**Table 1:** Correlation of inflammatory markers, with some anthropometric parameters in HIV seropositive pregnant participants with/without malaria and control participant.

Parameters	*R*	*p*
IL-6 vs IFN-*ɣ* (A) *n* = 32	0.479	0.006
TNF-*α* vs SPB (B) *n* = 30	−0.36	0.048
IFN-*ɣ* vs BMI (E)*****n* = 30	0.433	0.017

A = HIV seropositive pregnant women with malaria parasitemia, B = HIV seropositive pregnant women without malaria parasitemia, C = HIV seronegative pregnant women with malaria parasitemia, D = HIV seronegative pregnant women without malaria parasitemia, E = nonpregnant HIV seronegative women without malaria parasitemia. Control *r* = Pearson correlation coefficient. Correlation is significant when *p* is  ≤ 0.05.

## Data Availability

The data are available upon request to the corresponding author.
